# Determining Susceptibility and Potential Mediators of Resistance for the Novel Polymyxin Derivative, SPR206, in *Acinetobacter baumannii*

**DOI:** 10.3390/antibiotics13010047

**Published:** 2024-01-04

**Authors:** Jacinda C. Abdul-Mutakabbir, Nana Sakyi Opoku, Karen K. Tan, Peter Jorth, Victor Nizet, Hansel M. Fletcher, Keith S. Kaye, Michael J. Rybak

**Affiliations:** 1 Division of Clinical Pharmacy, Skaggs School of Pharmacy and Pharmaceutical Sciences, University of California San Diego, La Jolla, CA 92093, USA; vnizet@health.ucsd.edu; 2 Division of the Black Diaspora and African American Studies, University of California San Diego, La Jolla, CA 92093, USA; 3 Department of Basic Sciences, Loma Linda University School of Medicine, Loma Linda, CA 92374, USA; nsakyiopoku@students.llu.edu (N.S.O.); hfletcher@llu.edu (H.M.F.); 4 Department of Pharmacy, Loma Linda University Medical Center, Loma Linda, CA 92374, USA; karentan@llu.edu; 5 Department of Pathology and Laboratory Medicine, Cedars Sinai Medical Center, Los Angeles, CA 90048, USA; peter.jorth@cshs.org; 6 Department of Medicine, Rutgers University School of Medicine, New Brunswick, NJ 08854, USA; kk1116@rwjms.rutgers.edu; 7 Department of Pharmacy Practice, Anti-Infective Research Laboratory, Eugene Applebaum College of Pharmacy and Health Sciences, Wayne State University, Detroit, MI 48201, USA; m.rybak@wayne.edu; 8 Department of Medicine, Division of Infectious Diseases, Wayne State University, Detroit, MI 48201, USA

**Keywords:** *Acinetobacter baumannii*, polymyxin derivative, colistin, SPR206

## Abstract

With the increase in carbapenem-resistant *A. baumannii* (CRAB) infections, there has been a resurgence in the use of polymyxins, specifically colistin (COL). Since the reintroduction of COL-based regimens in treating CRAB infections, several COL-resistant *A. baumannii* isolates have been identified, with the mechanism of resistance heavily linked with the loss of the lipopolysaccharide (LPS) layer of the bacterial outer membrane through mutations in lpxACD genes or the *pmrCAB* operon. SPR206, a novel polymyxin derivative, has exhibited robust activity against multidrug-resistant (MDR) *A. baumannii*. However, there is a dearth of knowledge regarding its efficacy in comparison with other *A. baumannii*-active therapeutics and whether traditional polymyxin (COL) mediators of *A. baumannii* resistance also translate to reduced SPR206 activity. Here, we conducted susceptibility testing using broth microdilution on 30 *A. baumannii* isolates (17 COL-resistant and 27 CRAB), selected 14 COL-resistant isolates for genomic sequencing analysis, and performed time-kill analyses on four COL-resistant isolates. In susceptibility testing, SPR206 demonstrated a lower range of minimum inhibitory concentrations (MICs) compared with COL, with a four-fold difference observed in MIC_50_ values. Mutations in lpxACD and/or *pmrA* and *pmrB* genes were detected in each of the 14 COL-resistant isolates; however, SPR206 maintained MICs ≤ 2 mg/L for 9/14 (64%) of the isolates. Finally, SPR206-based combination regimens exhibited increased synergistic and bactericidal activity compared with COL-based combination regimens irrespective of the multiple resistance genes detected. The results of this study highlight the potential utility of SPR206 in the treatment of COL-resistant *A. baumannii* infections.

## 1. Introduction

*Acinetobacter baumannii* is an opportunistic, non-fermenting Gram-negative organism associated with high all-cause mortality rates [1,2]. Typically, *A. baumannii* manifests in nosocomial infections, with outbreaks identified in numerous countries and treatment settings globally [1,3]. *A. baumannii* shows a propensity for developing resistance against commonly used antimicrobials, including fluoroquinolones, aminoglycosides, and beta-lactams. The emergence of multidrug-resistant (MDR) *A. baumannii* isolates has led to a resurgence in using polymyxin agents, such as polymyxin B and colistin (COL, also known as polymyxin E) [4,5]. However, the increased use and the absence of optimal dosing for COL have resulted in reports of COL-resistant strains [2,5].

The mode of action for COL, a part of the polymyxin class of antimicrobials, involves an interaction with the polyanionic lipopolysaccharide (LPS) present within the bacterial outer membrane, leading to membrane destabilization [6]. Resistance of Gram-negative pathogens to COL is most commonly attributed to the modification of the lipid anchor of LPS, or lipid A. Consequently, mutations in the first three genes in the lipid A biosynthesis pathway, namely *lpxACD*, are frequently implicated as the basis for polymyxin resistance in *A. baumannii* [6,7]. Apart from *lpxACD* gene mutation, various studies have highlighted that modifications of the target LPS, driven by the addition of phosphoethanolamine moieties to lipid A through the *pmrCAB* operon, also play a role in *A. baumannii* COL resistance [8,9]. Of note, investigators have reported detection of mutations, specifically in the *pmrA* and *pmrB* genes, in COL- and carbapenem-resistant *A. baumannii* (CRAB) isolates [5,10]. When evaluated against MDR *A. baumannii* isolates, including those with *pmrA* and *pmrB* gene mutations, COL-based combination regimens (alongside other *A. baumannii*-active agents including meropenem, MEM, or minocycline, MIN) can be effective [11]. Nevertheless, conflicting data exists on whether these combinations are associated with improved patient outcomes or a reduction in the emergence of COL resistance [2,12]. Given the potentially detrimental spread of *A. baumannii* resistance to COL—a last-line agent—and dose-limiting toxicities associated with COL-based combination regimens, there is an urgent need to identify new agents to treat serious MDR *A. baumannii* infections.

SPR206, a novel polymyxin derivative, has shown robust activity against *A. baumannii* in both in vitro and in vivo studies [13,14]. While maintaining a similar pharmacophore to COL, SPR206 has undergone structural modifications to include a fatty acyl tail with an aryl chloride group-substituted aminobutyryl N-termini and a shortened nanopeptide cyclic core with L-Dap residues attached to the peptide ring [13,15]. These modifications have been attributed to a reduction in cytotoxicity and nephrotoxicity compared with COL, as observed in in vivo studies [16]. Additionally, in vitro studies have revealed that SPR206 has more potent activity compared with COL (nearly eight-fold lower MICs) when evaluated against MDR *A. baumannii* isolates [15]. Although these findings indicate the potential utility of SPR206 in MDR (including COL-resistant) *A. baumannii* infections, critical gaps remain. Namely, the potential mediators of SPR206 resistance need elucidation and whether these genes associated with *A. baumannii* COL resistance (i.e., *lpxACD* and/or *pmrA*, *pmrB*) exert the same impact on the novel polymyxin derivative remains unclear. Furthermore, there is a need to investigate whether similar or enhanced activity, comparative to COL, would be seen with SPR206 when tested in combination with other *A. baumannii*-active antimicrobials against MDR isolates.

In this study, our primary aims were to delineate the antibacterial activity of SPR206 against MDR *A. baumannii* isolates and to describe the effect of COL resistance on SPR206 susceptibility. The specific objectives of this study were to (i) evaluate the comparative activity of SPR206 and other *A. baumannii*-active agents against MDR *A. baumannii* isolates through minimum inhibitory concentration (MIC) testing, (ii) conduct genomic sequencing analysis to determine mutations present in 14 *A. baumannii* isolates (both COL-resistant and CRAB), and (iii) investigate and compare the in vitro synergistic activity, employing time-kill analysis (TKA), of COL and SPR206 alone as well as in combination regimens with other antimicrobials against four MDR A. *baumannii* isolates.

## 2. Results

### 2.1. Susceptibility Testing

Thirty isolates underwent MIC testing revealing that 17/30 (57%) were COL-resistant, with six isolates registering COL MIC values of 32 mg/L or higher [17]. In the MIC testing performed on *A. baumannii* strains, SPR206 inhibited the growth of (22/30) 73% of the isolates at concentrations of <2 mg/L and 83% (25/30) at concentrations of <4 mg/L. Two isolates exhibited an SPR206 MIC value of 32 mg/L. A four-fold increase in potency was shown in the MIC_50_ and MIC_90_ values of SPR206 compared with COL. Additionally, 87% (27/30) of the *A. baumannii* isolates demonstrated resistance to meropenem (MEM), with MIC values of ≥ 8 mg/L. Amikacin (AMK) and sulbactam (SUL) were largely ineffective in inhibiting *A. baumannii* growth, with 90% (27/30) presenting AMK MICs at >16 mg/L and with 77% (23/30) presenting SUL MICs at > 8 mg/L. Minocycline (MIN) and tigecycline (TGC) yielded more favorable MIC results, inhibiting *A. baumannii* growth at a MIN concentration of <4 mg/L in 77% (21/30) of the isolates and at a TGC concentration of <4 mg/L in 60% (18/30) of the isolates. Individual MIC values for each of the 30 tested isolates, alongside the MIC_50_ and MIC_90_ values of SPR206 and the comparative antimicrobials tested, can be found in Table 1.

### 2.2. Genomic Sequencing Analysis

Genomic sequencing was conducted on 14 COL-resistant and CRAB isolates collected from different geographical regions. Three were from Thailand (21%), one from Taiwan (7%), one from Israel (7%), and nine from Michigan (64%). The identified isolates exhibited elevated MICs and various resistance genes were detected, including aminoglycoside-modifying enzymes (notably APH (3′)-Vla) in all isolates. Beta-lactamases of classes A, C, and D, were present, with ADC-2 (an Acinetobacter-derived cephalosporinase) and bla_OXA-23_ in all isolates. Additionally, the endogenous presence of efflux pumps specific to the tetracycline agents (MIN and TGC) and the *lpxA* and *lpxC* genes was also confirmed in all *A. baumannii* isolates.

**Figure 1:**
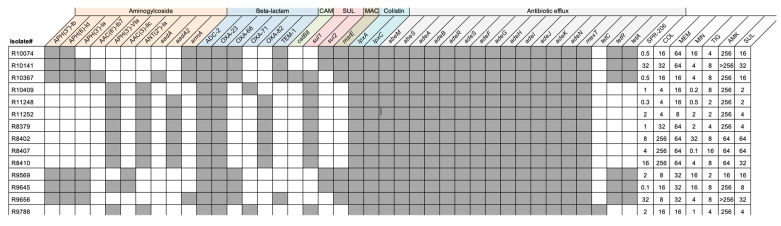
Resistance Genes Detected in 14 COL-resistant and CRAB isolates. Shown in Figure 1 are the resistance genes and MICs detected in the 14 COL-resistant and CRAB isolates. Gray boxes indicate the presence of given resistance genes in each isolate and white boxes indicate the gene was not detected. MICs for each isolate and antibiotic are indicated to the right.

Compared with a reference strain (CP043953.1), mutations in *pmrA* were detected, with missense single-nucleotide variants (SNV) encoding S119T and A144T mutations detected in *pmrA* for three of the 14 isolates (21%). Alterations in *pmrB* were more commonly detected with mutations detected in 13 of the 14 (93%) included isolates. Strain typing revealed that the isolates belonged to five unique clonal groups based on Oxford and Pasteur multilocus sequence typing (MLST) schemes for *A. baumannii* (Table 2). The most common sequence type among the strains was Pasteur ST2, followed by Pasteur ST3, Oxford ST106, ST195, and ST281. Figure 1 illustrates the mutations in the 14 sequenced isolates (COL-resistant and CRAB) and pmrCAB operon amino acid variations (and MLST sequence type) are provided in Table 2.

### 2.3. Time-Kill Analysis

**Figure 2:**
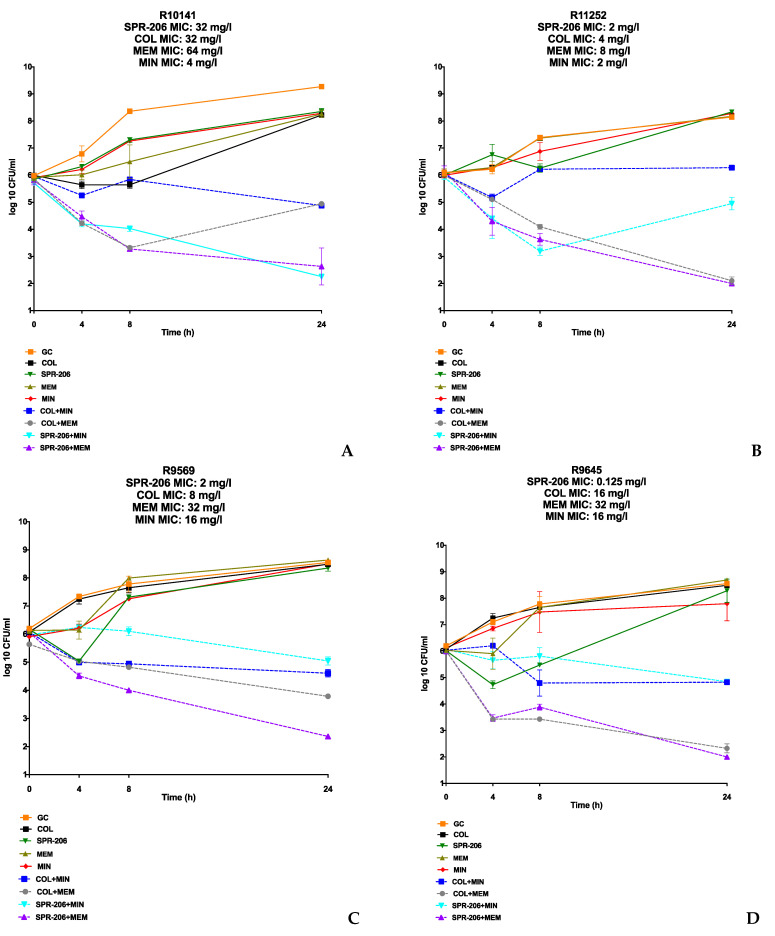
TKA Results of COL-resistant and CRAB Isolates. (**A**–**D**) are SPR206, COL, MEM, and MIN tested alone and in combination through TKA against COL-resistant and CRAB isolates. All individual agents were tested at 0.5× MIC or at the biological peak concentration (whichever was lower) alone and then in SPR-206- and COL-based combination regimens.

In the in vitro synergy evaluation of four COL-resistant and CRAB *A. baumannii* isolates, single agents did not sustainably reduce the bacterial burden over 24 h. However, the SPR206 + MEM combination displayed synergistic and bactericidal activity against all isolates, achieving an average −5.6 log_10_ CFU/mL reduction from the most active single agent. The SPR206 + MIN combination regimen was synergistic against all isolates exhibiting bactericidal activity against R10141. Despite elevated MICs for SPR206 and COL against R10141 with a detected *pmrB* mutation, SPR206-based combinations outperformed COL-based regimens with an average −5.25 log_10_ cfu/mL reduction compared with a −3.2 log_10_ cfu/mL reduction. COL + MEM showed synergistic activity against all isolates but achieved bactericidal activity against only two (R11252 and R9645). COL + MIN combinations were synergistic but yielded lower average log reductions (−2.78 log_10_ vs. −3.77 log_10_ reduction in cfu/mL from the most active single agent) compared with COL + MEM. Overall, the SPR206 plus MIN or MEM combinations demonstrated an average 3-log_10_ reduction in CFU/mL compared with the 2-log_10_ log reduction observed in the COL-plus-MEM or MIN combinations. The 24-h TKA results for the *A. baumannii* strains are shown in Figure 2.

## 3. Discussion

Given the escalating global threat of antimicrobial resistance, particularly in the context of COL-resistant CRAB infections, an increase in the utilization of COL is inevitable [1,18]. However, this heightened usage raises concerns about increased drug-related toxicities, including nephrotoxicity and neurotoxicity, and the continued dissemination of COL-resistant *A. baumannii* pathogens [1]. Identifying novel therapeutics becomes imperative to address this treatment gap. Our in vitro study highlights the robust activity of SPR206 against CRAB even in isolates with substantially elevated MICs to COL. Furthermore, our study suggests that traditional resistance mechanisms leading to elevated COL MICs, such as mutations in lpxACD or *pmrAB*, may not exert a similar impact on SPR206 susceptibility. Additionally, our results demonstrate enhanced activity when SPR206 is used in combination with other antimicrobials, even in the presence of resistance genes to either agent. This underscores the potential of SPR206 as a promising therapeutic option against COL-resistant *A. baumannii* infections.

In the last decade, several polymyxin derivatives have been developed, each featuring modifications to integral areas of the traditional polymyxin structure [13,19]. These modifications primarily involve changes to the N-terminal fatty acyl chain length, alterations in the hydrophobic domain of the COL, and substitutions of the Dab side chains and amino acids [20,21]. Of note, polymyxin derivative compounds with alterations in the hydrophobic domain of the N-terminus chain, such as SPR206, have demonstrated increased susceptibility compared with COL, a trend corroborated in our study [21]. SPR206 exhibited lower MICs in comparison to COL when evaluated against MDR *A. baumannii* isolates. Other studies have similarly reported lower MICs against MDR Gram-negative organisms compared with other polymyxin derivatives and polymyxin B. This improved activity may potentially be attributed to SPR206′s observed high LPS binding and permeabilization capabilities [21].

This heightened LPS binding and permeability capacity observed in SPR206 may contribute to its sustained activity even in the presence of COL resistance mediated by the loss of LPS genes (*lpxACD* and *pmrAB*) [22,23]. Each CRAB and COL-resistant isolate, characterized by the presence of multiple beta-lactamases, exhibited a mutation in either *lpxA*, *lpxC*, *pmrA*, or *pmrB.* This suggests that SPR206 may possess a higher barrier to resistance against LPS loss compared with COL. While *lpxACD* is more extensively studied in the context of COL resistance, the impact of the *pmrCAB* operon on *A. baumannii*-elevated MICs to COL is less understood [5,10,23,24]. It has been proposed that *pmrB* mutations could lead to the constitutive activation of *pmrA*, resulting in increased *pmrCAB* op-ron expression and COL resistance [25,26]. In our study, *pmrA* mutations were less common, while *pmrB* mutations were prevalent, potentially contributing to increased COL MICs. Nonetheless, SPR206 MICs remained relatively low, with 62% (9/14) of isolates having MICs at < 2 mg/L. In particular, isolates with elevated SPR206 MICs (>4 mg/L) exhibited *pmrB* mutations at A138T and/or amino acid substitutions, showing region-specific patterns. This regional variability is important for tailoring *A. baumannii* treatment strategies based on predominant clonal types in specific geographic regions [2,27,28]. Identifying molecular characteristics associated with elevated MICs to SPR206 is critical for informing the best practices in the treatment of *A. baumannii* infections.

In addition to SPR206 demonstrating retained susceptibility in the presence of multiple resistance genes, our study revealed similar and—in some cases—increased in vitro synergistic activity when SPR206 was combined with other antimicrobials compared with COL-based combination regimens. The enhanced membrane permeability attributed to polymyxins, including derivatives, has been hypothesized to facilitate the binding of drugs (such as MEM or MIN) when in combination irrespective of elevated MICs or gene mutations to either agent [2,11,29,30]. Given that SPR206 has demonstrated superior permeability compared with traditional polymyxins, this could explain why the SPR206-based combination regimens resulted in a greater reduction in CFU/mL compared with COL-based combinations [21]. Notably, the enhanced activity of MEM-containing combinations (SPR206 + MEM and COL + MEM) compared with MIN-containing combinations (SPR206 + MIN and COL + MIN) can be attributed to the fact that tetracyclines (MIN) are bacteriostatic agents, while carbapenems (MEM) are bactericidal [31,32]. This difference may influence the attenuated antimicrobial activity with MIN combinations compared with MEM combinations. Previous studies have also demonstrated increased activity with polymyxins in combination with carbapenems compared with combinations with tetracyclines, even in isolates with *pmrA* mutations [11,33].

Despite providing valuable insights about SPR206 activity, several limitations should be noted. First, only a select number of isolates underwent genomic sequencing analysis, potentially restricting the generalizability of the findings to a broader clinical applicability of the findings. Considering the epidemiological variations in *A. baumannii* infections, future studies should investigate SPR206 activity against prominent clonal ST types. Furthermore, polymyxin resistance in *A. baumannii* can be mediated through various factors, including other regulatory and effector mechanisms such as mutations in the *mcr-1* gene or in genes encoding OmpA family proteins. While each strain sequenced did have mutations in genes encoding OmpA family proteins (shown in Supplemental Table S1), the *mcr-1* gene was not detected in the selected sequenced strains [10,11]. Lastly, the MIC testing and TKA experiments were short-duration and used static concentrations, differing from humanized pharmacokinetic exposure conditions.

## 4. Materials and Methods

### 4.1. Bacterial Isolates

A total of 30 *A. baumannii* clinical isolates were included in the study; the isolates were representative of different geographical areas including Thailand, Israel, Taiwan, Michigan, and California. A portion (14/30) (46%) of the isolates were collected from patients who were enrolled in an NIH-funded clinical trial evaluating the treatment outcomes of extremely drug-resistant Gram-negative pathogen infections [34]. A total of 28/30 of the isolates were CRAB, indicated through the meropenem (MEM) MIC of ≥8 mg/L, and 15/30 were COL-resistant, indicated through the colistin (COL) MIC OF ≥4 mg/L [35]. To further present resistance mechanisms, genomic sequencing analyses were completed on 14 *A. baumannii* isolates, all of them being COL-resistant and CRAB isolates.

### 4.2. Antimicrobials

The comparator antibiotics that were utilized for susceptibility testing versus SPR206 in *A. baumannii* were as follows: MEM, COL, minocycline (MIN), sulbactam (SUL), amikacin (AMK), and tigecycline (TGC). MEM, COL, MIN, SUL, AMK, and TGC were purchased from Sigma Chemical Co. (St. Louis, MO, USA) and SPR206 was obtained from its manufacturer (SPERO Therapeutics Cambridge, MA, USA).

### 4.3. Susceptibility Testing

Susceptibility testing for COL, MEM, MIN, AMK, TGC, SUL, and MEM was performed for each strain in 96-well microtiter plates (Corning Costar^®^, obtained through Sigma-Aldrich^®^, Warren, MI, USA). Organism susceptibility (minimum inhibitory concentration, MIC) was evaluated through broth microdilution testing using cation-adjusted Mueller–Hinton broth (CAMHB, Difco, Detroit, MI, USA) supplemented with 25 mg/L Ca^2+^ and 12.5 µg/mL Mg^2+^ as stated in the Clinical and Laboratory Standards Institute (CLSI) guidelines. Freshly prepared Mueller–Hinton broth was used to prevent the oxidative degradation of TGC in aqueous solution and SUL was tested in combination with ampicillin (AMP) and supplemented at a 4:1 ratio. The microtiter, 96-well plates were incubated at 37 °C for 18–24 h before recording the results and minimum inhibitory concentration (MIC) reductions were measured using serial two-fold dilutions. *Escherichia coli* ATCC 25922 was used as the internal quality control strain.

### 4.4. Genomic Sequencing Analyses

Fourteen CRAB and COL-resistant isolates were selected to undergo whole genome sequencing (WGS). The total genomic DNA was extracted and used as input material for the library construction. DNA libraries were prepared using the Nextera XT™ library construction protocol and index kit (Illumina, San Diego, CA, USA) and sequenced on a MiSeq sequencer (Illumina). Libraries were multiplexed and sequenced with 100 base-pair (bp) paired end reads (PE100) on an Illumina NovaSeq 6000 (Illumina, San Diego, CA, USA). Samples were demultiplexed using bcl2fastq conversion software (v1.8.4) (Illumina, San Diego, CA, USA). Illumina genome sequencing reads were used for de novo genome assembly and annotation as well as re-sequencing analyses. The comprehensive genome analysis tool from the Bacterial and Viral Bioinformatics Resource Center (BV-BRC) was used to generate de novo assemblies and annotations for all genomes [36,37,38,39,40,41,42,43]. Beta-lactamase genes were identified through similarity to genes in the comprehensive antibiotic resistance database (CARD) [44]. Breseq (v0.37.1) was used for re-sequencing analysis to determine pmrA and pmrB single nucleotide variants [45]. Breseq was run in consensus mode to align sequencing reads according to the complete *A. baumannii* K09-14 reference genome sequence (Genbank accession CP043953.1).

### 4.5. Time-Kill Analyses

Time-kill analyses (TKAs) were performed against four isolates (CRAB and COL-resistant) in Mueller–Hinton broth (MHB) as growth media and each TKA was performed in duplicate for all antibiotic regimens to ensure reproducibility. In the TKA against the four *A. baumannii* isolates, each well was treated without a drug, SPR206, COL, MEM, MIN, SPR206 + MEM, SPR206 + MIN, COL + MEM, and COL + MIN, at a concentration of 0.5× MIC or the biological free peak (MEM fCmax at 30 mg/L per 1 g q 8 h dosing, COL fCmax at 2 mg/L per 4.5 million IU q 12 h dosing, and MIN fCmax at 8 mg/L per 200 mg IV q 12 h dosing), utilizing whichever was lower [32,46,47]. The experiments were conducted at a starting inoculum of ~1 × 10^6^ for each isolate and were conducted in a shaker incubator at 37 °C for 24 h and aliquots of 0.1 mL were obtained from each well at the 0-, 4-, 8-, and 24-h time intervals.

The samples were serially diluted in 0.9% normal saline according to the appropriate concentrations and plated using automatic spiral plating (EasySpiral Pro Intersciences, Worburn, MA, USA); then, the plates were incubated at 37 °C for 24 h before colony enumeration using an automated colony counter (Scan 1200, Interscience Laboratories Inc., Woburn, MA, USA). The time-kill curves were made by plotting mean colony counts remaining from duplicate experiments against each time point using Prism^®^ (v10.1.2)(Graphpad Software, San Diego, CA, USA). Bactericidal activity was defined as ≥3 log_10_ CFU/mL reduction from baseline and synergistic activity was defined as a ≥2 log_10_ CFU/mL reduction from the most active single agent. Antagonistic activity was defined as a ≥2 log_10_ CFU/mL decrease in killing from the most active single agent.

## 5. Conclusions

In summary, this study reveals that SPR206 has robust activity against *A. baumannii* isolates, including those characterized by CRAB and COL-resistance. Importantly, SPR206 MIC values consistently outperformed COL MIC results when evaluated against *A. baumannii*. Traditional mechanisms of polymyxin-resistance, mediated through LPS loss and the modification of *lpxACD* or the *pmrCAB* operon, may not significantly impact SPR206 activity. Further research is warranted to assess the viability of SPR206 as a broadly applicable treatment option for MDR *A. baumannii* infections and its diverse potential mediators of resistance.

## Figures and Tables

**Table 1:** Broth microdilution susceptibility testing results (mg/L).

Isolate Number	Geographical Location	SPR-206	COL	MEM	MIN	TIG	AMK	SUL
J105	Loma Linda Medical Center, Loma Linda, CA	1	1	16	0.5	0.5	64	64
J104	Loma Linda Medical Center, Loma Linda, CA	1	0.5	32	0.5	1	64	64
J109	Loma Linda Medical Center, Loma Linda, CA	0.25	0.5	8	2	1	8	32
J108	Loma Linda Medical Center, Loma Linda, CA	1	1	32	0.5	1	64	64
J110	Loma Linda Medical Center, Loma Linda, CA	0.5	1	8	1	1	32	32
J101	Loma Linda Medical Center, Loma Linda, CA	0.25	1	32	2	1	64	64
J103	Loma Linda Medical Center, Loma Linda, CA	1	1	32	1	1	64	64
J108	Loma Linda Medical Center, Loma Linda, CA	1	2	32	2	1	64	64
J106	Loma Linda Medical Center, Loma Linda, CA	1	0.5	4	4	2	64	64
J115	Loma Linda Medical Center, Loma Linda, CA	1	2	8	0.5	2	32	8
J112	Loma Linda Medical Center, Loma Linda, CA	0.25	2	8	0.5	2	16	16
J107	Loma Linda Medical Center, Loma Linda, CA	0.5	2	32	1	2	64	16
R11248	Detroit Medical Center, Detroit MI	0.25	4	16	0.5	2	256	2
J113	Loma Linda Medical Center, Loma Linda, CA	1	4	4	1	2	32	16
J102	Loma Linda Medical Center, Loma Linda, CA	1	4	32	1	2	32	64
R11252	Detroit Medical Center, Detroit MI	2	4	8	2	2	256	4
J111	Loma Linda Medical Center, Loma Linda, CA	1	4	2	2	2	32	32
R9569	Detroit Medical Center, Detroit MI	2	8	32	16	2	16	16
J114	Loma Linda Medical Center, Loma Linda, CA	0.5	1	16	0.5	4	32	16
R9788	Assaf Harofeh Medical Center, Israel	2	16	16	1	4	256	4
R10074	Siriraj Hospital, Bangkok, Thailand	0.5	16	64	16	4	256	16
R8379	Corewell Health Detroit, MI	1	32	64	2	4	256	4
R10409	Siriraj Hospital, Bangkok, Thailand	1	4	16	0.25	8	256	2
R9656	Siriraj Hospital, Bangkok, Thailand	32	8	32	4	8	>256	32
R10367	Chaung Gung Medical Hospital, Taiwan	0.5	16	16	4	8	256	16
R9645	Siriraj Hospital, Bangkok, Thailand	0.125	16	32	16	8	256	8
R10141	Corewell Health Royal Oak, MI	32	32	64	4	8	>256	32
R8410	Corewell Health Royal Oak, MI	16	256	64	4	8	64	32
R8402	Corewell Health Royal Oak, MI	8	256	64	32	8	64	64
R8407	Corewell Health Royal Oak, MI	4	256	64	0.125	16	64	64
MIC_50_		1	4	32	1	2	64	16
MIC_90_		8	32	64	4	8	256	64

Shown in Table 1 are the individual MIC, MIC_50_, and MIC_90_ values for the 30 *A. baumannii* isolates evaluated against *A. baumannii*-active antimicrobials.

**Table 2:** *pmrA* and *pmrB* amino acid variations for 14 COL-resistant and CRAB isolates.

Isolate	Geographical Location	Sequence Type (Oxford/Pasteur)	*pmrA* Amino Acid Variations	*pmrB* Amino Acid Variations
**R10074**	Siriraj Hospital, Bangkok, Thailand	ST195 (O) ST2 (P)	WT	A138T
**R10141**	DMC, Detroit MI	Not assigned	WT	A138T
**R10363**	Chaung Gung Medical Hospital, Taiwan	ST129 (P)	S119T	A227V
**R10409**	Siriraj Hospital, Bangkok, Thailand	ST106 (O) ST3 (P)	S119T	P360Q
**R11248**	DMC, Detroit MI	ST281 (O) ST2 (P)	WT	L239V
**R11252**	DMC, Detroit MI	ST2 (P)	WT	A142T
**R8379**	Corewell Health, Royal Oak MI	ST2 (P)	WT	L239V
**R8402**	Corewell Health, Royal Oak MI	ST2 (P)	WT	D37G, Q43L
**R8407**	Corewell Health, Royal Oak MI	ST2 (P)	WT	D37G, Q43L
**R8410**	Corewell Health, Royal Oak MI	ST281 (O) ST2 (P)	WT	D37G, Q43L
**R9569**	DMC, Detroit MI	ST2 (P)	WT	WT
**R9645**	Siriraj Hospital, Bangkok, Thailand	ST2 (P)	WT	P233T
**R9656**	Siriraj Hospital, Bangkok, Thailand	ST195 (O) ST2 (P)	WT	A138T, R263L
**R9788**	Assaf Harofeh Medical Center, Israel	ST106 (O) ST3 (P)	A14T, S119T	T187P, P360Q

Shown in Table 2 are the geographical location and *pmrA* and *pmrB* amino acid variations compared with the reference strain (CP043953) for the 14 COL-resistant and CRAB isolates. MLST strain typing according to Oxford (O) or Pasteur (P) schemes for *A. baumannii*.

## Data Availability

Data are contained within the article and supplementary materials.

## Supplementary Materials

The following supporting information can be downloaded at: https://www.mdpi.com/article/10.3390/antibiotics13010047/s1, Supplementary Table S1—Mutations in Genes Encoding for OmpA family Proteins for 14 COL-resistant and CRAB isolates.
