# Supplementary material for: Determining Susceptibility and Potential Mediators of Resistance for the Novel Polymyxin Derivative, SPR206, in Acinetobacter baumannii
Source: Antibiotics (Basel). 2024 Jan 4;13(1):47. doi: 10.3390/antibiotics13010047 (PMC10812597; doi:10.3390/antibiotics13010047)
Supplement: Supplementary file 1 [file antibiotics-13-00047-s001.zip › antibiotics-2796066-supplementary.pdf]

Supplemental Table S1. Mutations in Genes Encoding for OmpA family Proteins for 14 COL-resistant and CRAB isolates

position	mutation	R10074	R10141	R10363	R10409	R11248	R11252	R8379	R8402	R8407	R8410	R9569	R9645	R9656	R9788	annotation	gene	description
514,005	C→T			100%												A12T (GCA→ACA)	*F3P16_02520* ←	OmpA family protein
622,556	2 bp→CT			100%	100%										100%	coding (113-114/1071 nt)	*F3P16_03030* →	OmpA family protein
622,584	2 bp→CG			100%	100%										100%	coding (141-142/1071 nt)	*F3P16_03030* →	OmpA family protein
622,587	C→G			100%	100%										100%	N48K (AAC→AAG)	*F3P16_03030* →	OmpA family protein
622,596	4 bp→TTCA	100%	100%			100%	100%	100%	100%	100%	100%	100%	100%	100%		coding (153-156/1071 nt)	*F3P16_03030* →	OmpA family protein
622,707	2 bp→T			100%	100%										100%	coding (264-265/1071 nt)	*F3P16_03030* →	OmpA family protein
622,711	+G			100%	100%										100%	coding (268/1071 nt)	*F3P16_03030* →	OmpA family protein
622,961	C→G			100%	100%										100%	A173G (GCT→GGT)	*F3P16_03030* →	OmpA family protein
622,971	3 bp→CTT			100%	100%										100%	coding (528-530/1071 nt)	*F3P16_03030* →	OmpA family protein
622,981	G→A			100%	100%										100%	E180K (GAG→AAG)	*F3P16_03030* →	OmpA family protein
2,501,958	C→T				100%										100%	V35I (GTA→ATA)	*F3P16_11815* ←	OmpA family protein
2,502,003	C→T				100%										100%	A20T (GCA→ACA)	*F3P16_11815* ←	OmpA family protein
2,674,297	C→T				100%										100%	D400N (GAT→AAT)	*F3P16_12590* ←	OmpA family protein
2,674,639	T→C	100%	100%	100%		100%	100%	100%	100%	100%	100%	100%	100%	100%		N286D (AAC→GAC)	*F3P16_12590* ←	OmpA family protein
2,674,759	C→T				100%										100%	V246I (GTT→ATT)	*F3P16_12590* ←	OmpA family protein
2,674,861	C→T				100%										100%	A212T (GCT→ACT)	*F3P16_12590* ←	OmpA family protein
2,674,903	T→C	100%	100%	100%	100%	100%	100%	100%	100%	100%	100%	100%	100%	100%	100%	T198A (ACA→GCA)	*F3P16_12590* ←	OmpA family protein
2,675,347	T→C				100%										100%	I50V (ATA→GTA)	*F3P16_12590* ←	OmpA family protein
2,884,210	A→C				100%										100%	L233V (TTA→GTA)	*F3P16_13615* ←	OmpA family protein
2,884,336	A→G	100%	100%	100%	100%	100%	100%	100%	100%	100%	100%	100%	100%	100%	100%	S191P (TCA→CCA)	*F3P16_13615* ←	OmpA family protein
2,884,448	A→T	100%	100%	100%		100%	100%	100%	100%	100%	100%	100%	100%	100%		D153E (GAT→GAA)	*F3P16_13615* ←	OmpA family protein
2,884,497	2 bp→CG	100%	100%	100%	100%	100%	100%	100%	100%	100%	100%	100%	100%	100%	100%	coding (409-410/780 nt)	*F3P16_13615* ←	OmpA family protein
2,884,500	G→T	100%	100%	100%	100%	100%	100%	100%	100%	100%	100%	100%	100%	100%	100%	A136E (GCA→GAA)	*F3P16_13615* ←	OmpA family protein
2,884,518	T→G	100%	100%	100%		100%	100%	100%	100%	100%	100%	100%	100%	100%		E130A (GAA→GCA)	*F3P16_13615* ←	OmpA family protein
2,884,524	A→C				100%										100%	L128W (TTG→TGG)	*F3P16_13615* ←	OmpA family protein
2,884,527	A→G				100%										100%	V127A (GTT→GCT)	*F3P16_13615* ←	OmpA family protein
2,884,553	2 bp→TC				100%										100%	coding (353-354/780 nt)	*F3P16_13615* ←	OmpA family protein
2,884,597	T→C				100%										100%	I104V (ATT→GTT)	*F3P16_13615* ←	OmpA family protein
2,884,624	G→T				100%										100%	H95N (CAC→AAC)	*F3P16_13615* ←	OmpA family protein
2,884,642	T→C				100%										100%	I89V (ATC→GTC)	*F3P16_13615* ←	OmpA family protein

Supplemental Table S1. *Cont.*

2,884,648	2 bp→AT				100%										100%	coding (258-259/780 nt)	*F3P16_13615* ←	OmpA family protein
2,884,753	T→C	100%	100%	100%		100%	100%	100%	100%	100%	100%	100%	100%	100%		I52V (ATT→GTT)	*F3P16_13615* ←	OmpA family protein
2,884,819	C→T	100%	100%	100%		100%	100%	100%	100%	100%	100%	100%	100%	100%		E30K (GAA→AAA)	*F3P16_13615* ←	OmpA family protein
2,884,824	T→G				100%										100%	Q28P (CAA→CCA)	*F3P16_13615* ←	OmpA family protein

Shown in Supplemental Table S1 are mutations found in genes encoding for OmpA family proteins in the 14 sequenced COL-resistant and CRAB isolates.
